# Evaluating the long-term effects of income assistance for material hardship among families with children

**DOI:** 10.1016/j.ssmph.2024.101700

**Published:** 2024-07-21

**Authors:** Molly Grant, Kane Meissel, Dan Exeter, Susan M.B. Morton

**Affiliations:** a Faculty of Education and Social Work, University of Auckland, Auckland, New Zealand; b School of Population Health, University of Auckland, Auckland, New Zealand; c Faculty of Health, University of Technology Sydney, Sydney, Australia

## Abstract

**Background:**

As a key aspect of poverty, material hardship describes day-to-day struggles in affording necessities. In explorations of policy initiatives that mitigate material hardship, evidence suggests direct income support can be effective in alleviating hardship. However, research investigating the long-term effects of income supports is limited, and it remains uncertain as to how benefit receipt may mitigate material hardship for families with children across time.

**Methods:**

To explore the associations between income assistance and material hardship longitudinally, we utilised data from four waves of the *Growing Up in New Zealand* birth-cohort study (*n* = 5964), where family experiences were tracked between birth and adolescence. The variables of interest included mother-reported receipt of income benefits and material hardship from when the children were aged 9-months, 54-months, 8-years, and 12-years. Multilevel logistic regression analysis was employed to examine the associations between benefit receipt and material hardship in the short- and long-term. Interaction terms between benefit receipt and time were incorporated in the modelling to determine whether receiving benefits corresponded with significant changes in the likelihood of experiencing material hardship at specific time points.

**Results:**

The key results suggest that benefit receipt in early childhood reduced the likelihood of experiencing material hardship at subsequent time points at least until adolescence, when controlling for key sociodemographic characteristics.

**Conclusion:**

These findings provide insight into the associations between early childhood income supports and reductions in the likelihood of experiencing material hardship in the long-term, to emphasise the potential for such interventions to have longstanding effects. By elucidating the associations between benefit receipt and subsequent material hardship, this research gives direction for policy interventions and timely support for families.

## Highlights

• Effects of benefit receipt on material hardship.
• Findings show positive associations between benefit receipt and concurrent hardship.
• Early childhood benefits reduced material hardship likelihood in adolescence.

## 1 Introduction

Families living in material hardship face challenges in providing for their basic needs (Beverly, 2001). As such, material hardship describes the inability to afford basic necessities such as safe housing, nutritious meals, and adequate healthcare (Mayer & Jencks, 1989). Prior research highlights the ramifications of material hardship for families, with evidence linking these experiences to negative child health outcomes (Frank et al., 2010), internalising and externalising behaviour problems in childhood (Zilanawala & Pilkauskas, 2012), and maternal factors such as elevated risk of depression (Heflin & Iceland, 2009). Evidently, the effects of hardship extend beyond economic limitations to various facets of family life including physical, mental, and emotional wellbeing and development. Consequently, identifying initiatives that can both prevent and reduce material hardship holds potential to foster improvements for the wellbeing of families with children.

### 1.1 Determinants of material hardship

In this paper we focus on material hardship, describing the inability to afford necessities across household indicators including food, clothing, utilities, and other essential costs (Beverly, 2001). Factors at the family-level can directly influence material hardship. For example, household economic factors such as low income and housing tenure can limit a family's ability to purchase essential resources, leading to instances of material hardship (Cai et al., 2023; Morrissey et al., 2020; Perry, 2022). Equally, situations that raise the cost of necessities or limit job opportunities can increase the risk of material hardship (Levy, 2015). Certain sociodemographic characteristics are also associated with an increased likelihood of facing material hardship including sole-parent households (Pilkauskas et al., 2012), minoritised ethnic groups (Ankuda et al., 2021), those with lower levels of education (Neckerman et al., 2016), and those with poorer health (Heflin & Butler, 2013). These demographic associations highlight the presence of structural inequalities in *who* experiences hardship, where certain groups face disproportionate levels of material hardship (Munari et al., 2021). This uneven distribution in who experiences material hardship underscores the importance of implementing policies and initiatives aimed at addressing material hardship. Such efforts are essential for creating more equitable environments so more families can live free from material hardship. Accordingly, to target material hardship, income assistance programmes have been implemented to help reduce experiences of material hardship and promote economic stability for families (Pilkauskas, 2023).

Income assistance programmes have received noticeable focus from researchers (e.g., Pilkauskas, 2023) and policymakers (e.g., Perry, 2022) as viable initiatives to address material hardship at a policy-level. These programmes are typically provided by governmental organisations to help cover basic household expenses such as food, clothing, and housing (Sullivan et al., 2008). The nature and extent of income support schemes varies widely across different countries and according to individual circumstances, but examples of these programmes include tax credits, one-off cash transfers, unemployment benefits, and pensions. Although material hardship does not necessarily coincide with income poverty (Bradshaw & Finch, 2003; Neckerman et al., 2016), robust evidence indicates that when families receive direct cash transfers, this income ‘boost’ can help mitigate hardship (Collyer et al., 2022; Livermore et al., 2015; Parolin et al., 2021; Shaefer & Gutierrez, 2013). For example, a study by Parolin et al. (2021) showed expansions to an income assistance programme decreased the likelihood of hardship. In their study, difference-in-differences estimates were used to examine the effects of the 2021 expansion to the Child Tax Credit (CTC) for families in the US. Their results suggested that receiving the CTC led to a significant 14-percentage point decrease in the likelihood of facing food hardship compared with the pre-treatment hardship rate. These findings align with the conceptual framework proposed by Gershoff et al. (2003) for programmes and policies addressing child poverty. This framework explains income support programmes which directly augment family income can be pivotal strategies for poverty reduction by assisting families to acquire basic necessities. We draw on this conceptual framework in this study to highlight initiatives aimed at creating economic opportunities for families as mechanisms through which sustainable solutions to material hardship can be realised. Accordingly, the present study focuses on benefit receipt in the form of means-tested benefits that provide direct cash transfers to families. This focus contributes to the extension of research in this important area by exploring the specific associations of direct cash transfers for households experiencing material hardship, to provide targeted insights for stakeholders seeking to address policy alleviation.

### 1.2 Sustained effects of benefit receipt

Leveraging longitudinal data—where measures of material hardship and benefit receipt are collected from the same families over multiple intervals—offers an opportunity to observe the interplay between benefit receipt and fluctuations in hardship. As such, longitudinal research enables comprehensive explorations of the pathways linking these variables, to unpack the potential effectiveness of benefits in targeting hardship mitigation in the short- and long-term. Indeed, studies examining benefits and material hardship have often utilised datasets with longitudinal designs (e.g., Collyer et al., 2022; Kondratjeva et al., 2022; Reichman et al., 2005). This prior research has largely focused on relatively short-term effects, typically within a year of benefit receipt, to explain changes in family material circumstances following in the immediate aftermath of receiving income support (e.g., Kalil et al., 2002; Kondratjeva et al., 2021; Reichman et al., 2004). These insights are essential for understanding the immediate implications of benefits for recipient families. However, there remains a notable gap in this literature concerning long-term effects as few studies have examined broader time spans to assess sustained implications of benefits for families. Addressing this gap is imperative for understanding the enduring effects of benefits, thereby informing policymakers about the efficacy of benefit programs in mitigating hardship and fostering long-term economic stability for families.

The long-term effects of income benefit receipt refer to the continuing implications for families who receive financial support. The effects of income assistance programmes have the potential to extend beyond immediate relief and go on to influence economic stability, health, and other areas of wellbeing, both intra- and inter-generationally. Positive long-term effects of benefits have been observed for maternal health (Jones et al., 2022), child health (Braga et al., 2020), household savings (Jones & Michelmore, 2018), debt reduction (Shaefer et al., 2013), and improvements in wages (Kuka & Shenhav, 2020). However, the long-term effects of benefit receipt are relatively unexamined for material hardship. One study provides an example of an exception where the effects of sanctions to a cash assistance programme for single-parent families were examined (White, 2023). While White (2023) observed cumulative effects of sanctions, by which a greater number of sanctions over time increased the probability of material hardship, beyond their research, limited attention has been paid to investigating the effects of benefits for material hardship longitudinally. Such explorations would provide insights for policymakers about how income benefits may contribute to reducing material hardship over time. For instance, if receiving benefits leads to short-term relief but fails to contribute to the reduction of hardship in the long-term, this would suggest a need for more comprehensive anti-poverty strategies. Examining the effects of benefit receipt and the potential association with material hardship over time is also important for understanding if there are certain time points during which benefits may be particularly helpful. For example, infancy is a time where parents often take leave from work which can result in a loss of income. Income benefits may provide much-needed financial support during this period of reduced income and assist families in staving off material hardship immediately and in the long-term. For example, Kuka and Shenhav (2020) found single mothers in the US who benefited from tax credits in their child's first year of life had higher earnings in the long-run than those who received benefits 3–6 years later. Therefore, benefits early on might support families to navigate through this phase and regain stability over time. Accordingly, further investigation into benefit receipt, the timing of this receipt, and long-term associations with hardship are warranted.

### 1.3 Aotearoa New Zealand context

The context of Aotearoa New Zealand (NZ) can provide valuable insights into benefit receipt and material hardship. Current research has explored the effects of benefit receipt for poverty within varied contexts including the US (e.g., Collyer et al., 2022; Kondratjeva et al., 2022; Reichman et al., 2005), continental Europe (e.g., Guio et al., 2022), and low-middle income countries (e.g., Hjelm et al., 2017). However, it is also necessary to understand the policy landscapes and hardship experiences in NZ. Extrapolating findings from other contexts to NZ may offer some relevance where there are similarities in income assistance programmes. For example, both the US and 10.13039/501100001562NZ offer support for low-income families through cash transfers and social services (Perry, 2022; Reichman et al., 2005). However, disparities in benefit levels, coverage, and socioeconomic contexts indicate that material hardship experiences, and the susceptibility of these experiences to respond to benefits, may vary. For example, for many households in the US, a substantial portion of income is allocated to healthcare costs and insurance (Dieleman et al., 2020). In contrast, 10.13039/501100001562NZ operates a universal publicly funded healthcare system where households may spend relatively little on healthcare services, as these are free of charge or heavily subsidised (Gauld, 2020). These contextual differences illustrate one example of variation in the cost of necessities between nations and underscore the importance of examining the NZ-specific landscape of material hardship. Preliminary evidence from Gray and colleagues (2021) highlights the link between benefit receipt and hardship within the NZ context. Their mixed-methods study revealed that the 248 participants who experienced sanctions on their benefits commonly resorted to reducing spending on necessities and relying on support networks to cope financially. However, beyond this study there is limited research examining the association between benefit receipt and material hardship. Further research within this context is warranted.

### 1.4 The current study

This study aims to contribute to the discourse on poverty alleviation strategies. We utilise longitudinal data from NZ families, gathered across four time points when children were in infancy through to adolescence. The primary focus of this research is to investigate the effects of means-tested benefits on material hardship across time. Delving into the long-term associations allows for the identification of sustained effects, as well as shifts in the significance and strength of associations over the four time points. Such analyses can contribute to the broader context of this research area by elucidating whether observed associations are consistent or subject to change across time. This research offers a novel contribution to this field in that instances of material hardship are tracked longitudinally. Few studies have examined the temporal dynamics of material hardship over time (e.g., Thomas, 2022) and it is essential to explore this further to provide robust research which explores the nuances of varied measurement approaches and contexts. Therefore, a secondary aim of this research is to provide preliminary insights into how material hardship unfolds for families across childhood. The research question underpinning this study is.

- How is benefit receipt associated with material hardship for families with children across four discrete time points, from infancy through to adolescence?

## 2 Data and methods

### 2.1 Data source

Data from the longitudinal birth-cohort study, *Growing Up in New Zealand* (GUiNZ), were utilised for this study and full details about the study design can be found in prior work (e.g., Morton et al., 2013). Briefly, the study recruited 6822 expectant mothers residing in three regions of NZ with anticipated delivery dates between 2009 and 2010. The subsequent cohort of 6853 children constitute a diverse sample that is broadly representative of all national births occurring between 2007 and 2010 (Morton et al., 2015). Major data collection waves at different stages of the children's development have been conducted including before birth, at 9-months, 54-months, 8-years, and 12-years, with supplementary age-specific data gathered between these main phases. Ethical approval for the study was granted from the 10.13039/100009647Ministry of Health *Northern Y Regional Ethics Committee* (NTY/08/06/055) in 10.13039/501100001562NZ.

### 2.2 Measures

#### 2.2.1 Outcome variables: material hardship

Data relating to material hardship were utilised from four different data collection waves—the 9-month, 54-month, 8-year, and 12-year intervals. The primary caregivers of the children provided responses to a set of five binary items about material hardship at each time point, indicating whether or not they had experienced each hardship in the past 12 months. These items focused on limitations to paying for food, clothing, utilities, and other everyday costs. At each time point, the five items were aggregated into an index, ranging from 0 to 5, before determining an optimal threshold on this scale to categorise families as either ‘not living in hardship’ or ‘living in hardship’. We operationalised material hardship as a binary variable to align with common academic and policy objectives, aiming for a clear distinction between families experiencing hardship and those facing less acute forms of adversity or none at all (Alkire & Foster, 2011; Sen, 1976). To mitigate the subjectivity of selecting a suitable threshold, we adopted Notten and Kaplan's (2022) method for setting poverty thresholds. This approach used additional data, such as participants' reports of economic strain, to evaluate different thresholds and identify the one most likely to accurately differentiate participants experiencing hardship from those who were not. Accordingly, families were organised into respective categories of ‘not living in hardship’ or ‘living in hardship’ at each time point. The derivation of the material hardship variables is further described in prior work (Grant, Meissel, & Exeter, 2024).

#### 2.2.2 Explanatory variables: benefit receipt

At each of the four time points, mothers provided information about the various sources of income their households had received over the past year. This information included whether or not the household had received income assistance through government benefits (i.e., participants selected ‘yes’ or ‘no’ to receipt of various forms of government assistance). Therefore, for analyses, benefit receipt was operationalised as categorical variables at each wave: benefit-recipients and non-recipients. This operationalisation aligns with previous studies of benefit receipt where a binary variable was also employed (e.g., Collyer et al., 2022). Benefit recipients were receiving at least one type of government administered income-tested support including unemployment support, sole parent assistance, disability and sickness allowances, accommodation supplements, student allowance, training incentives, and other benefits. In 10.13039/501100001562NZ, family tax credits are also a form of income support for families with dependent children in the low-to-middle income distribution (Inland Revenue, 2023). While family tax credits fall under the umbrella of means-tested benefits these differ from other benefits in that they are available to the majority of working families with children. Accordingly, recipients of family tax credits who were not receiving any other assistance were not included in the benefit receipt group for analyses. As a robustness check, we categorised families into three groups to recognise households receiving multiple support programmes may differ to those receiving one form of assistance (Wu et al., 2022). These categories included: recipients of one benefit, recipients of two or more benefits, and non-recipients. This approach reflects the need for some families to access multiple, rather than a single, programmes.

#### 2.2.3 Covariates: sociodemographic variables

Sociodemographic variables were predominantly sourced from the antenatal data collection wave. Despite holding potential to vary over time, we treated all covariates as time-invariant due to large amounts of missing data at the later time points, and as the primary focus of this study is on the relationship between benefit receipt over time and material hardship, rather than on the covariates themselves. The covariates included.

• *Mother's highest level of education* was determined from self-reported information and organised into categories: no secondary school qualification; secondary school/National Certificate of Educational Achievement (NCEA) levels 1–4; diploma/trade certificate/NCEA levels 5–6, bachelor's degree; and a higher degree.
• *Housing tenure* describes each family's housing arrangement as: owns home; private rental; public rental; or other.
• *Family structure* was organised into two categories to describe the household structure at the antenatal time point: sole parent household; two parent household.
• *Number of children* denotes the count of children in the family unit and is treated as a continuous variable ranging from 0 to 10 children.
• *Maternal age at birth* was calculated from the mother's date of birth and the child's birthdate and categorised into the following categories: younger than 25 years; 25–35 years; older than 35 years.
• *Maternal general health* was self-reported by the mother and response options ranged from poor to excellent (scale: 0–4).
• *Maternal ethnicity* was classified into the following major ethnic groups in alignment with the Statistics New Zealand Level 1 ethnic groupings: Māori (Indigenous people of NZ); Pacific Peoples; Asian; Other; and European (Statistics New Zealand, 2020). However, at the antenatal wave, there were a large proportion of cases with missing ethnicity data (9.40%, *n* = 569). To retain as many cases as possible in the analyses, we chose to utilise ethnicity data from the 12-year data collection wave. Then, where cases were missing ethnicity data at this time point, data collected at previous time points were used to ‘fill in’ the data. This resulted in *n* < 10 missing cases.

### 2.3 Data preparation

The following subsections detail the steps for readying the data for analyses.

#### 2.3.1 Missing data

Given the longitudinal nature of the study design, only families with two or more waves of material hardship data were considered for the analytic sample (*n* = 6073). Additionally, for benefit receipt, cases with more than two missing time points were removed due to the large portion of missingness (1.79%, *n* = 109). This resulted in an analytic sample of *n* = 5964 families. However, the number of families with material hardship data at each wave still varied over time within this sub-sample (see Table 1). Notably, there were large proportions of missing data for the 8-year and 12-year intervals, with greater than 20.0% missing data at both time points, due to increased attrition in the sample at these time points. In terms of the missing data at the case-level, 21.53% of families (*n* = 1284) had one time point with missing hardship data, and 13.62% of families (*n* = 813) had two time points with missing data. The overall missing data for material hardship prompted the decision to employ multi-level modelling (see section 2.4.1 for further details), where cases with missing dependent variable values could be retained by leveraging the available information in the process of estimating the model parameters.

**Table 1:** Material hardship data (n = 5964).

Time point	Available material hardship data		Missing data	
	*n*	%	*n*	%
**9-months**	5862	98.29	102	1.71
**54-months**	5869	98.41	95	1.59
**8-years**	4731	79.33	1233	20.67
**12-years**	4484	75.18	1480	24.82

*Note.* Percents are row percentages.

Relating to benefit receipt, 2250 cases (37.05%) had at least one missing value across the four time points. Little's MCAR test yielded a significant result (*p* < 0.05), suggesting the missing data were not missing completely at random (MCAR) and removing cases by listwise deletion could risk biased parameter estimates. Although the absence of data could possibly be attributed to unobserved mechanisms (i.e., missing not at random), we chose to operate under the assumption of missing at random (MAR). This decision was grounded in the context of family studies, where it is reasonable to expect other variables likely account for missing values (Acock, 2005). In this sense, the missingness is likely to depend on other variables and assuming MAR in this study is plausible as the missingness likely relates to factors beyond benefit receipt. Accordingly, an imputation strategy to fill in the missing data was undertaken that included auxiliary variables. The auxiliary variables were chosen based on the potential correlation with benefit receipt, aiming to help account for any observed associations between the missingness and the variable, and to reduce the nonresponse bias (Enders, 2022). Imputation was performed using multiple imputation by chained equations (MICE) using the *mice* package in RStudio (van Buuren & Groothuis-Oudshoorn, 2011), and informed by the sociodemographic variables of maternal education, maternal age, and family structure at child's birth. Additionally, auxiliary variables including area-level deprivation at each wave informed the imputation. MICE was considered appropriate as it has been shown to produce more reliable results compared with other methods for dealing with missing data such as listwise deletion and mean substitution (Enders, 2022). A total of 100 imputed datasets were generated (with maximum iterations of 20) and results were pooled in the analyses.

#### 2.3.2 Analytic sample

In Table 2, the sociodemographic profile of the families included in the analytic sample is shown (see column two). These sociodemographic characteristics were compared to those of the full GUiNZ sample at baseline (see column three, *n* = 6822). To assess how those included in the current study differed to the baseline sample by sociodemographic characteristics, logistic regression was employed. The findings from this analysis indicated a lower inclusion rate for families residing in medium–high deprivation areas compared with low deprivation, mothers identifying as a non-European ethnicity, mothers having lower levels of education at their child's birth, and younger mothers. Given these sample variations, it is important to recognise that the results from the present study may not be generalisable to the GUiNZ sample at baseline or the broader adolescent population of NZ. However, the prevalence of material hardship observed in this study is similar to material hardship trends obtained in national-level official statistics (Statistics New Zealand, 2023).

**Table 2:** Sample characteristics and comparisons to baseline sample.

	Current sample	Baseline sample	Response rate	Logistic model	
	*n*	N	(%)	Odds ratio	*p*-value
**Total**	5964	6822	87.42	–	–
**Socioeconomic deprivation (NZDep2006)**					
Low deprivation (scores 1–3)	1593	1694	94.04	1.00	Ref.
Medium deprivation (scores 4–7)	2258	2486	90.83	0.63	***
High deprivation (scores 8–10)	2110	2640	79.92	0.25	***
Missing data	<10	<10	–	–	–
**Maternal ethnicity**					
Māori (Indigenous people)	1136	1355	83.91	0.21	***
Pacific	773	990	78.08	0.14	***
Asian	828	990	83.64	0.20	***
Other	175	232	75.43	0.12	***
European	3050	3172	96.15	1.00	Ref.
Missing data	<10	83	–	–	–
**Mother education at birth**					
No secondary school qualification	360	491	73.32	0.21	***
Secondary school/NCEA 1-4	1325	1627	81.44	0.33	***
Diploma/Trade cert/NCEA 5-6	1822	2082	87.51	0.52	***
Bachelor's degree	1432	1539	93.05	1.00	Ref.
Higher degree	1009	1064	94.83	1.37	ns
Missing data	16	19	–	–	–
**Mother age at birth**					
Younger than 25 years	1008	1289	78.20	0.40	***
25–35 years	3348	3725	89.88	1.00	Ref.
Older than 35 years	1605	1735	92.51	1.39	**
Missing data	<10	73	–	–	–

*Note.* *** <0.001; ** <0.01; ns = non-significant at *p* < 0.05; ref. = reference group. NZDep2006 is an area-deprivation index, formulated using data from the 2006 NZ census and determined by a set of neighbourhood deprivation items (Salmond et al., 2007). The deprivation scores are divided into deciles, with the tenth decile representing the most deprived 10% of neighbourhoods. *n* < 10 are supressed in accordance with data access guidelines.

### 2.4 Data analysis

All analyses were performed using RStudio Software (version 2022.07.0.548; RStudio Team, 2022). The following sub-section details the approach used to understand how benefit receipt was associated with material hardship.

#### 2.4.1 Multi-level modelling

As there were multiple time points of material hardship data nested within each family in the sample (i.e., at 9-months, 54-months, 8-years, and 12-years), multilevel logistic regression models (MLM), via adaptive Gauss-Hermite quadrature with ten points, were employed to understand how benefit receipt was associated with material hardship while controlling for the correlated error among the residuals (Austin & Merlo, 2017; Nestler, 2023). We used two-level models to allow the repeated measures of material hardship (level 1) to be nested within families (level 2). The MLM uses maximum likelihood estimators to accommodate incomplete data on the dependent variable (Enders, 2023).

We started by fitting the simplest MLM using the *lme4* package in RStudio (Bates et al., 2009), which accounted for the family effects on material hardship, but without the inclusion of any predictor variables. This ‘null’ model contained only an intercept, no fixed explanatory variables, and can be expressed in the form of:

$$\log\left(\frac{\pi ij}{1-\;\pi ij}\right)=\beta_{0}+u_{0j}$$

The equation explains the log-odds of experiencing hardship (πij) at each time point i, within each family j. The intercept $\left(\beta_{0}\right)$ captures the average log-odds for the whole sample, while the random effect $\left(u_{0j}\right)$ accounts for the variability between families in experiencing hardship.

Given we were interested in material hardship over time, we used MLM to examine how time was associated with living in hardship, when accounting for the variability introduced by the family-level random effects. The following equation describes the relationship between the log-odds of material hardship occurring, and the predictor variable of time, while accounting for the group-level variation captured by the random effect:

$$\log\left(\frac{\pi ij}{1-\;\pi ij}\right)=\beta_{0}+\beta_{1}{time}_{ij}+u_{0j}$$

Time was treated as a categorical variable, with each time point represented as a discrete instance (i.e., 9-month, 54-month, 8-year, and 12-year). Therefore, we were able to ascertain, compared with the 9-month wave (used as the reference point) if there was a significant increase or decrease in the log-odds of experiencing material hardship at subsequent waves or not. The predictor variables (benefit receipt and covariates) were then added to the model, and finally, we added interaction terms between benefits and time. This final model can be expressed as:

$$\log\left(\frac{\pi ij}{1-\;\pi ij}\right)=\beta_{0}+\beta_{1}{time}_{ij}$$

$$+\beta_{2}{benefit9months}_{ij}+\beta_{3}{benefit54months}_{ij}+\beta_{4}{benefit8years}_{ij}+\beta_{5}{benefit12years}_{ij}$$

$$+\beta_{6}{time*benefit9months}_{ij}+\beta_{7}{time*benefit54months}_{ij}$$

$$+\beta_{8}{time*benefit8years}_{ij}+\beta_{9}{time*benefit12years}_{ij}$$

$$+\beta_{10}{covariate1}_{ij}+\beta_{11}{covariate2}_{ij}+\beta_{12}{covariate3}_{ij}+\beta_{13}{covariate4}_{ij}+\beta_{14}{covariate5}_{ij}+\beta_{15}{covariate6}_{ij}+\beta_{16}{covariate7}_{ij}$$

$$+u_{0j}$$

The purpose of this final model was to ascertain if benefit receipt at a specific time point was associated with a significant increase or decrease in the log-odds of experiencing material hardship at the 54-month, 8-year, or 12-year stages compared to the 9-month wave. This approach enabled us to delve into the nuanced temporal aspects of the relationships between benefits and material hardship. For interpretability, we converted the estimated regression coefficients to odds ratios by exponentiating the log-odds.

## 3 Results

### 3.1 Descriptive statistics

Table 3 details the number of families who were living in hardship, those not living in hardship, and the corresponding levels of missing data at each time point. This table provides insight into the proportion of families who were experiencing hardship. At each time point, the majority of families were not experiencing hardship. Further descriptive investigation revealed a large proportion of families (79.16%; *n* = 4721) did not experience material hardship at any of the four time points. However, one in five families (20.84%, *n* = 1243) experienced at least one instance of material hardship in their child's first 12 years of life. Additionally, 7.98% (*n* = 476) experienced two or more spells of hardship. A small number of families experienced hardship across all four time points (*n* = 25).

**Table 3:** Material hardship at each wave (n = 5964).

Time point	In hardship		Not in hardship		Missing data	
	*n*	%	*n*	%	*n*	%
**9-months**	604	10.13	5258	88.16	102	1.71
**54-months**	518	8.69	5351	89.72	95	1.59
**8-years**	418	7.01	4313	72.32	1233	20.67
**12-years**	374	6.27	4110	68.91	1480	24.82

*Note.* Percents are row percentages.

For benefit receipt, Table 4 details the proportion of families receiving a means-tested benefit at each time point. The 9-month time point had the highest proportion of families receiving benefits, with one in every four families receiving assistance (25.80%, *n* = 1539). By the 12-year time point, one in ten families were receiving a benefit (9.71%, *n* = 579).

**Table 4:** Estimates of benefit receipt at each wave (n = 5964).

Time point	Benefit-recipient		Non-recipient	
	*n*	%	*n*	%
**9-month**	1539	25.80	4425	74.20
**54-month**	1585	26.58	4379	73.42
**8-year**	630	10.56	5334	89.44
**12-year**	579	9.71	5385	90.29

*Note.* Percents are row percentages. Proportions were calculated by taking the averages values from across the multiple imputed datasets, in accordance with Rubin's rules (Rubin, 1987).

### 3.2 MLM of benefit receipt and material hardship

The results from the MLM are presented in the sub-sections below.

#### 3.2.1 Interpretation of the null two-level model

After fitting the simplest MLM (one with no predictor variables), we found that the odds ratio of experiencing hardship was estimated as 0.02 (*p* < 0.001), indicating that most families were not living in hardship at baseline (see Table 5, Model 1). The variance at the family-level was 5.85, which was non-zero and therefore, indicated that controlling for the family-level variance was sensible. After comparing the likelihood ratio statistic from the null model with the corresponding single-level model (i.e., without the level 2 random effects), the test statistic equated to 1521 with one degree of freedom, providing strong evidence that the between-family variance was non-zero. This result further indicated that multi-level modelling was preferable over single-level modelling. We also examined the intraclass correlation coefficient, which indicated that 64% of the variance in the material hardship values was explained by between-family differences (and 36% explained by within-family differences over time). Taken together, these explorations of the null two-level model indicated it was important to consider the nested structure of the data.

**Table 5:** Estimated odds ratios of experiencing material hardship.

**Intercept**	Odds Ratio (95% Confidence Intervals)			
	Model 1	Model 2	Model 3	Model 4
	**0.02 (0.02**–**0.02)**	**0.02 (0.02**–**0.03)**	**0.01 (0.00**–**0.01)**	**0.01 (0.01**–**0.01)**
**PREDICTORS**				
**Time *(ref.* 9-months*)***				
54-months		**0.76 (0.65**–**0.89)**	**0.75 (0.63**–**0.89)**	**0.64 (0.50**–**0.83)**
8-years		0.92 (0.78–1.09)	0.99 (0.83–1.19)	0.82 (0.63–1.07)
12-years		0.89 (0.75–1.05)	0.92 (0.76–1.11)	**0.63 (0.47**–**0.84)**
**Benefit receipt**				
Benefit 9-months *(ref. no receipt)*			**1.90 (1.53**–**2.35)**	**2.77 (2.05**–**3.74)**
Benefit 54-months *(ref. no receipt)*			**1.63 (1.33**–**2.01)**	1.16 (0.86–1.57)
Benefit 8-years *(ref. no receipt)*			**1.92 (1.50**–**2.47)**	1.22 (0.84–1.79)
Benefit 12-years *(ref. no receipt)*			**2.04 (1.57**–**2.63)**	**1.51 (1.03**–**2.22)**
**COVARIATES**				
**Ethnicity *(ref. European)***				
Māori			**1.66 (1.30**–**2.13)**	**1.65 (1.29**–**2.11)**
Pacific			**4.46 (3.42**–**5.81)**	**4.41 (3.37**–**5.77)**
Asian			1.02 (0.74–1.40)	1.00 (0.73–1.38)
Other			1.28 (0.73–2.26)	1.26 (0.71–2.24)
**Education *(ref. Bachelor's degree)***				
No secondary school			**2.66 (1.80**–**3.94)**	**2.60 (1.75**–**3.87)**
Secondary school/NCEA 1–4			**2.38 (1.77**–**3.20)**	**2.35 (1.74**–**3.16)**
Diploma/Trade cert./NCEA 5–6			**2.03 (1.54**–**2.70)**	**2.01 (1.51**–**2.67)**
Higher degree			0.96 (0.67–1.40)	0.97 (0.66–1.40)
**Housing tenure *(ref. Own home)***				
Private rental			**1.87 (1.53**–**2.29)**	**1.86 (1.52**–**2.28)**
Public rental			**3.51 (2.55**–**4.84)**	**3.51 (2.54**–**4.86)**
Other			0.99 (0.54–1.83)	0.98 (0.53–1.81)
**Family structure *(ref. Two-parent)***				
Sole parent household			**1.50 (1.14**–**1.96)**	**1.49 (1.13**–**1.96)**
**Maternal age at birth *(ref. 25–35 years)***				
Younger than 25 years			0.79 (0.62–1.02)	0.78 (0.61–1.00)
Older than 35 years			**0.79 (0.63**–**0.99)**	**0.79 (0.63**–**0.99)**
**Number of children**			1.46 (1.34–1.59)	**1.46 (1.34**–**1.59)**
**Maternal general health**			0.81 (0.74–0.88)	**0.81 (0.74**–**0.88)**
**INTERACTIONS**				
Benefit 9-months (ref.*Time 9-months*)*				
*Time 54-months				**0.56 (0.39**–**0.82)**
*Time 8-years				**0.54 (0.36**–**0.82)**
*Time 12-years				**0.61 (0.40**–**0.93)**
Benefit 54-months (ref. *Time 9-months*)*				
*Time 54-months				**2.11 (1.44**–**3.09)**
*Time 8-years				1.32 (0.87–2.00)
*Time 12 years				1.45 (0.93–2.25)
Benefit 8-years (ref. *Time 9-months*)*				
*Time 54-months				1.43 (0.90–2.27)
*Time 8-years				**3.14 (1.95**–**5.05)**
*Time 12-years				1.51 (0.91–2.51)
Benefit 12-years (ref. *Time 9-months*)*				
*Time 54-months				1.06 (0.66–1.69)
*Time 8-years				1.10 (0.66–1.83)
*Time 12-years				**3.07 (1.88**–**4.99)**

*Note.* Bolded odds ratio indicates statistical significance at *p* < 0.05. Ref. = reference group. Continuous scores were converted to z-scores.

#### 3.2.2 Adding the explanatory variables

***Time.*** We then included time as an explanatory variable in the model (see Table 5, Model 2). The odds ratios for the 8-year and 12-year time points were not significantly different from the 9-month time point. These findings can be interpreted as the prevalence rates of material hardship in the sample being relatively similar across these three timepoints, after accounting for the variability introduced by the family-level random effects. However, compared with the 9-month time point, the 54-month time point was significantly associated with decreased odds of experiencing material hardship in the overall sample (*OR* = 0.76, *p* < 0.001), suggesting a lower prevalence of hardship in the sample at this time point.

***Benefit receipt and covariates.*** When the benefit variables and covariates were added to the model (see Model 3), we observed that recipient families were significantly more likely to experience hardship compared to non-recipients, reflecting the targeted nature of these benefits. This result was observed at each discrete time point. For example, families who received a benefit at the 8-year wave were nearly twice as likely to experience hardship overall compared to those not receiving a benefit at the 8-year wave (*OR* = 1.92, *p* < 0.001). Additionally, a higher incidence of hardship was significantly associated with mothers who were of Māori and Pacific ethnic identity, those with an education level lower than a university degree, those living in a private or public rental, sole parent households, and those with more children. Mothers with better general health were at decreased odds of experiencing hardship (*OR* = 0.81, *p* < 0.001).

#### 3.2.3 Interaction effects

Model 4 is the fully adjusted model which includes the covariates, explanatory variables, and interactions between benefits and time. Adding this extra complexity to the MLM changed the significance and interpretation of some of the individual predictors, revealing a more nuanced understanding of the associations with the outcome variable. For time, the 54-month and 12-year time points were significant in the final model (*OR* = 0.64, *p* < 0.001; *OR* = 0.63, *p* < 0.01). These results suggest, compared with the 9-month time point, there were decreased odds of experiencing hardship in the full sample at these points, meaning there was a significantly lower rate of hardship when accounting for the covariates and interaction effects.

When the time interaction terms were integrated into the model, no notable changes in the significance or effect sizes were observed across the covariates. These findings suggest the associations between the covariates and material hardship remained consistent, independent of the specific timing of benefit receipt. This stability implies, regardless of when families received benefits, the effect of key demographic characteristics (e.g., ethnicity, education) on material hardship remained relatively constant over the data collection period.

For benefit receipt, families who received a benefit at 9-months and 12-years were more likely to experience hardship compared to non-recipients (*OR* = 2.77, *p* < 0.001; *OR* = 1.51, *p* < 0.05). However, benefits at 54-months and 8-years was non-significant. The lack of statistical significance during these periods indicates the inclusion of the interaction effects attenuated the effects of benefit receipt at these time points. However, these results need to be interpreted in conjunction with the interaction effects.

**9-month *interaction effects***. In Model 4, benefit recipients at 9-months were more likely to experience hardship during their child's first 12 years than non-recipients. However, the estimates from the interaction terms suggest that, over time, benefit receipt at 9-months was associated with a significant decreased likelihood of being in hardship at each subsequent time point. Put differently, early recipients saw greater reductions in the likelihood of experiencing hardship in the latter three waves than did non-recipients. For example, at the 54-month time point these families were at reduced odds of experiencing hardship (*OR* = 0.56, *p* < 0.01). The effect size was attenuated slightly at the 12-year time point (*OR* = 0.61, *p* < 0.05) and yet, there remained an enduring trend of decreased odds of hardship among those families who received benefits early on. Robustness checks align with these findings in that those receiving one type of benefit were at a significant decreased likelihood of being in hardship at each subsequent time point (see Supplementary Information). However, no statistically significant findings were observed for families receiving two or more benefits, potentially reflecting more complexities in the circumstances of those receiving multiple forms of assistance.

**54-month *interaction effects***. Unlike the benefit receipt at the 9-month stage, benefits received at 54-months did not seem to exert the same influence on the likelihood of facing material hardship at subsequent intervals. Specifically, while benefit receipt at 54-months was not significantly associated with increased odds of experiencing hardship overall (*OR* = 1.16, *p* > 0.05), the interaction effects suggest that families receiving benefits at the 54-month stage were twice as likely to be living in hardship during this period (compared to the 9-month stage; *OR* = 2.11, *p* < 0.001). Yet, benefit receipt at 54-months did not significantly affect the likelihood of experiencing material hardship at the later time points (8-year, *OR* = 1.32, *p* > 0.05; 12-year, *OR* = 1.45, *p* > 0.05).

***8-year interaction effects.*** For the interactions at the 8-year wave, benefit recipients were over three times more likely to experience hardship during this period than they were at the 9-month wave (*OR* = 3.14, *p* < 0.001). However, benefit receipt at 8-years did not significantly increase or decrease the likelihood of experiencing material hardship at the 12-year wave (*OR* = 1.51, *p* > 0.05).

***12-year interaction effects.*** Families who received benefits at the 12-year time point were more likely to experience hardship overall (*OR* = 1.51, *p* < 0.01), and were at increased odds of experiencing hardship at 12-years, compared to the 9-month time point (*OR* = 3.07, *p* < 0.001).

## 4 Discussion

This study explored the long-term effects of income assistance for material hardship using longitudinal data gathered from families with children. Data spanning from infancy through to early adolescence allowed for an assessment of material hardship and benefit receipt, where families were provided with income support to help meet basic costs. We outline five key discussion points that serve as a framework for interpreting the findings from this study in relation to broader research in this field.

### 4.1 Immediate associations between benefit receipt and hardship

In Model 3, the empirical findings showed a positive association between benefit receipt and material hardship. At each of the four time points, families were at heightened risk of hardship if they received a benefit. For example, families who received benefits at the 9-month time point had an increased risk of experiencing material hardship, compared with those who did not receive benefits at 9-month. These findings seem somewhat counterintuitive in that benefits should work to reduce hardship, and not be associated with an increased likelihood of hardship. However, we consider these findings likely to reflect the nature of correlational research (rather than interventional research), in that those receiving a benefit were more likely to be in hardship. Previous research has also observed positive associations between income support and material hardship (Wu & Eamon, 2010). One explanation for these observations is that families in need may only become eligible for benefits when they are already experiencing hardship. Thus, the receipt of benefits would coincide with periods of hardship. In this case, the observation that families accessing benefits were more likely to be living in hardship signifies an alignment between support systems and those in need of financial support. Therefore, these findings should not be interpreted as receiving a benefit exacerbating material hardship, rather, benefits were likely serving the intended purpose by being received by the families who were facing financial challenges at the time of receipt.

### 4.2 Long-term effects of benefit receipt

Using MLM, we were able to understand the long-term associations of income assistance programmes for material hardship. Notably, our findings showed a decreased likelihood of hardship among early benefit recipients over time. Specifically, recipients at 9-months were more likely to experience hardship than non-recipients, but across subsequent waves, their likelihood of facing hardship reduced by almost half. Benefits provided early on in childhood seem to have reduced the likelihood of future hardship. Importantly, the effect size of early benefits was maintained almost eight years later, and only attenuated slightly at the 12 year time point. This consistency of effect size likely demonstrates the enduring effects of early benefits and showcases a sustained influence up to 12 years later. The ramifications of these findings are important, not only for families but also for policymakers in that the timely delivery of assistance programmes may have effects over an extended period of time. As noted by McInnis et al. (2023) in their report of the intergenerational effects of public assistance for poverty, recognising the prolonged positive effects of income assistance programmes is essential so as not to underestimate the returns on the investments in these programmes.

The long-term findings from this study contribute to the existing literature in several ways. Firstly, these results align with the conceptual framework proposed by Gershoff et al. (2003), suggesting income assistance can help mitigate material hardship for families by augementing income. Furthermore, these findings add to the body of evidence finding income assistance programmes mitigate hardship (Collyer et al., 2022; Kondratjeva et al., 2021; Parolin et al., 2021; Wu et al., 2022). Previous work found short-term effects of benefits in reducing medical hardship (Kondratjeva et al., 2021; Wu et al., 2022). Our results extend on these findings in that a composite measure of material hardship was assessed. Therefore, our results show that benefit receipt may also be effective in mitigating material hardship as a broader construct. Secondly, previous research has highlighted the lasting effects of benefit programmes for maternal economic factors such as maternal debt reduction (Shaefer et al., 2013) and improvements for maternal wages (Kuka & Shenhav, 2020). The results from the current study extend on these findings by demonstrating another area where there are likely long-lasting intra-generational effects of benefits—for material hardship. By explaining the temporal trend wherein early benefit recipients exhibited a decreasing likelihood of hardship nearly 12 years later, this study highlights the potential lasting effects of benefits in mitigating hardship for families with children.

The importance of benefits for families during infancy warrants further discussion. Our ability to observe long-term effects was focused on early childhood where there were three subsequent time periods of data available after the 9-month period. Indeed, we found long-lasting effects of receipt at this 9-month time point. These results may be explained by infancy being a stage where families have reduced income due to time off work. Receiving a benefit at this early developmental stage may have served as a buffer, mitigating financial strain before parents returned to paid employment, thus, staving off later hardship. Such conclusions align with related research by Kuka and Shenhav (2020) who found single mothers who received tax credits in infancy had higher earnings in the long-run than those who received benefits when their child was older. Income assistance benefits early on might support families through economic challenges and help them to regain stability over time. However, it is important to clarify that our interpretation of the findings here do not advocate for brief periods of benefits as being sufficient to prevent future hardship. This cautionary statement stems from the evidence of cessation to benefits having negative implications for hardship (Kalil et al., 2002; Lee et al., 2004; Livermore et al., 2015). Consequently, while short-term benefits might offer hardship relief in the long-term, premature discontinuation could lead to deeper hardship.

### 4.3 Assistance does not equate to being “stuck” in hardship

When considering the interaction effects in the MLM, we found no evidence to suggest benefit receipt *increased* the likelihood of hardship in the future. While the estimates suggested that recipient families were more likely to be living in hardship at the time of receipt, our findings did not indicate any link between receipt and an increased likelihood of hardship at subsequent periods. From these results we can infer that, while families required supports during financially challenging times, the support they received did not confine them to a perpetual state of need. These conclusions are supported by previous research promoting the efficacy of assistance programmes in mitigating hardship (Collyer et al., 2022; Kalil et al., 2002; Kondratjeva et al., 2021, 2022; Reichman et al., 2004, 2005; Wu et al., 2022). Accordingly, benefits likely serve as a temporary bolster to assist families in navigating difficulties and eventually regaining stability. These findings challenge any notion that benefit programmes create a situation where recipients are unable to transition out of hardship nor that these families can never experience upward mobility. Instead, income assistance programmes likely offer support for families in securing basic necessities and can help facilitate their path towards improved circumstances.

### 4.4 Temporal dynamics of hardship

The findings from the MLM also offered preliminary insights into how material hardship unfolds for families with children. The results from the fully adjusted model indicated the prevalence of material hardship fluctuated across time, with decreased odds observed at the 54-month and 12-year waves, compared with the 9-month time point. Evidently, there were temporal shifts in the occurrence of hardship, which aligns with prior research indicating material hardship is not necessarily stable across time (Thomas, 2022). Our descriptive results suggested one in five families experienced material hardship at some point in their child's first 12-years, albeit the proportion of families in hardship at each discrete wave was less than one in ten. These results likely reflect fluctuations in circumstances, where many families transitioned in and/or out of hardship over the four time points. While the specific patterns of transition—that is, the extent to which families moved in and out of hardship—were not examined in this study, future work could explore the trajectories of hardship in more detail to understand the nuances of how material hardship is experienced over time. Such work would complement the findings from this study by contributing to the limited research on the persistence and transitions associated with material hardship longitudinally.

### 4.5 Sociodemographic predictors of hardship

While this research was not specifically focused on subgroup experiences of hardship, by including sociodemographic covariates in the modelling we were able to gain insights into who was more likely to experience hardship. We observed that families with certain sociodemographic features were at increased likelihood of hardship. As is consistent with previous work (e.g., Perry, 2022), sole-parent households, those with Māori and Pacific ethnic identities, lower educated, and renters were more likely to experience hardship. Mothers with better general health were less likely to experience hardship, also aligning with previous research (Heflin & Butler, 2013). Furthermore, we observed stability in the associations between the covariates and material hardship when adding the interaction terms to the modelling. This consistency is noteworthy as it suggests that demographic characteristics held their effects on hardship, independent of when families received benefits. These findings speak to the pervasiveness of structural inequities in that wellbeing outcomes continue to be unequally experienced based on sociodemographic characteristics—suggesting systemic failure in providing equitable support which is perpetuating unequal experiences. Yet, all families have the fundamental right to live free from hardship. These findings underscore the need for prioritised interventions, specific to the needs of these groups, in addressing sociodemographic disparities and alleviating material hardship.

### 4.6 Limitations and future work

While our findings reveal significant associations between benefit receipt and material hardship, the conclusions do not establish a causal relationship. There are likely unmeasured factors that could explain the relationship between benefits and hardship over time that were not accounted for. Despite this, the results here provide new evidence about the long-term implications of benefits for material hardship, and therefore, offer an important foundation in understanding the prolonged effects of income assistance. Future work should continue to explore the temporal dynamics of benefit receipt through longitudinal experimental designs to offer insights into the causal pathways underlying the relationship between benefits and material hardship.

Another limitation is that societal and economic shifts across time were not accounted for in the modeling. Factors such as fluctuations in economic conditions, shifts in unemployment statistics, and changes in benefit sizes were not accounted for in our analyses. Future studies should consider incorporating a wider array of variables to offer further understanding of the nuanced interplay between benefit receipt and hardship. Furthermore, we were unable to quantify the size of benefits received as this information was not available in the datasets. Consequently, our research does not provide an indication of the size of benefits needed to assist families in covering the costs of basic necessities. We also acknowledge that different types of benefits (e.g., sole parent support versus disability support) vary in nature, and that we have not included a separate examination of these benefits. Accordingly, we have not provided information as to how different benefits are associated with material hardship over the long-term. However, the aim of this study was to provide an initial exploration into the enduring effects of benefit receipt, and therefore, further explorations were out of scope.

Although our study indicates positive long-term implications of early benefit receipt, these benefits did not eliminate hardship in the sample. There is ongoing need for further exploration and innovative approaches aimed at supporting families to exit from hardship. Continued research efforts should delve into additional strategies, focusing on comprehensive solutions that target the root causes of financial distress among vulnerable families. In saying this, our findings point to the dangers of eliminating income assistance programmes in that there is risk hardship will persist for many families without such supports. Income assistance programmes remain pivotal, serving as a safety net crucial in supporting families to navigate adversity.

Finally, caution should be exercised when extrapolating these findings to full GUiNZ cohort and broader population of NZ. The differences observed between the baseline GUiNZ sample and the participants in this study may mean the outcomes of this study do not necessarily translate to the broader population. Nonetheless, it is worth noting the prevalence of material hardship observed in this study is similar to material hardship trends obtained from national-level official statistics (Statistics New Zealand, 2023), lending credibility to the findings.

### 4.7 Conclusion

Ultimately, this research contributes to the ongoing discourse on poverty alleviation strategies and underscores the importance of early interventions. Using comprehensive longitudinal data from families with children, we explored the immediate and long-term effects of benefit receipt at four time points in relation to material hardship across infancy through to adolescence. Our findings revealed important insights—while benefit receipt increased the likelihood of material hardship, receipt in infancy significantly reduced the likelihood of future instances of hardship. These results shed light on the potential positive effects of benefits being visible over long durations. Prioritised support during early life is likely a feasible initiative to help mitigate persistent hardship and help foster sustained wellbeing for families with children.

## Funding

This research did not receive any specific grant from funding agencies in the public, commercial, or not-for-profit sectors.

## Data statement

Data are available via application to *Growing Up in New Zealand.*

## Declaration of generative AI in scientific writing

During the preparation of this work the authors used ChatGPT to improve readability in some sections. After using this tool, the authors reviewed and edited the content as needed and take full responsibility for the content of the publication.

## Ethical statement

Ethical approval for the *Growing Up in New Zealand* study was granted from the Ministry of Health Northern Y Regional Ethics Committee (NTY/08/06/055) in Aotearoa New Zealand.

## CRediT authorship contribution statement

**Molly Grant:** Writing – review & editing, Writing – original draft, Methodology, Formal analysis, Conceptualization. **Kane Meissel:** Writing – review & editing, Supervision, Methodology, Conceptualization. **Dan Exeter:** Writing – review & editing, Supervision, Methodology, Conceptualization. **Susan M.B. Morton:** Writing – review & editing, Supervision.

## Declaration of competing interest

None.

## Data Availability

The authors do not have permission to share data.
