# Supplementary material for: Evaluating the long-term effects of income assistance for material hardship among families with children
Source: SSM Popul Health. 2024 Jul 21;27:101700. doi: 10.1016/j.ssmph.2024.101700 (PMC11327433; doi:10.1016/j.ssmph.2024.101700)
Supplement: Multimedia component 1 [file mmc1.docx]

**Evaluating the Long-Term Effects of Income Assistance for Material Hardship Among Families with Children**

## Supplementary Information

**Table S1**

*Robustness Check for Estimated Odds Ratios of Experiencing Material Hardship*

|  | | **Odds Ratio (95% Confidence Intervals)** | | | |
| --- | --- | --- | --- | --- | --- |
|  | **Model 1** | | **Model 2** | **Model 3** | **Model 4** |
| **Intercept** | **0.02 (0.02–0.03)** | | **0.03 (0.02–0.03)** | **0.01 (0.00–0.01)** | **0.01 (0.00–0.01)** |
| **PREDICTORS** |  | |  |  |  |
| **Time *(ref. 9-months)*** |  | |  |  |  |
| 54-months |  | | **0.77 (0.66–0.90)** | **0.75 (0.63.–0.88)** | **0.64 (0.50.–0.83)** |
| 8-years |  | | 0.91 (0.77–1.07) | 0.99 (0.83–1.19) | 0.85 (0.65–1.11) |
| 12-years |  | | 0.88 (0.74–1.04) | 0.92 (0.76–1.10) | **0.64 (0.48–0.85)** |
| **Benefit receipt** |  | |  |  |  |
| Benefit 9-months *(ref. no receipt)* |  | |  |  |  |
| One benefit |  | |  | **1.73 (1.37–2.16)** | **2.74 (1.99–3.76)** |
| Two or more benefits |  | |  | **2.10 (1.53–2.88)** | **2.57 (1.60–4.13)** |
| Benefit 54-months *(ref. no receipt)* |  | |  |  |  |
| One benefit |  | |  | **1.38 (1.10–1.74)** | 1.04 (0.74–1.46) |
| Two or more benefits |  | |  | **2.36 (1.77–3.15)** | **1.54 (1.01–2.33)** |
| Benefit 8-years *(ref. no receipt)* |  | |  |  |  |
| One benefit |  | |  | **1.85 (1.40–2.44)** | 1.29 (0.85–1.97) |
| Two or more benefits |  | |  | **2.04 (1.36–3.04)** | 0.99 (0.52–1.87) |
| Benefit 12-years *(ref. no receipt)* |  | |  |  |  |
| One benefit |  | |  | **1.68 (1.27–2.23)** | 1.31 (0.85–2.01) |
| Two or more benefits |  | |  | **2.83 (1.87–4.28)** | **2.05 (1.08–3.88)** |
| **COVARIATES** |  | |  |  |  |
| **Ethnicity *(ref. European)*** |  | |  |  |  |
| Māori |  | |  | **1.62 (1.27–2.07)** | **1.61 (1.26–2.06)** |
| Pacific |  | |  | **4.45 (3.41–5.80)** | **4.38 (3.35–5.73)** |
| Asian |  | |  | 1.01 (0.73–1.38) | 0.98 (0.72–1.36) |
| Other |  | |  | 1.33 (0.76–2.32) | 1.31 (0.74–2.31) |
| **Education *(ref. Bachelor’s degree)*** |  | |  |  |  |
| No secondary school |  | |  | **2.62 (1.77–3.87)** | **2.57 (1.73–3.82)** |
| Secondary school/NCEA 1–4 |  | |  | **2.34 (1.74–3.14)** | **2.32 (1.72–3.12)** |
| Diploma/Trade cert./NCEA 5–6 |  | |  | **1.98 (1.50–2.62)** | **1.97 (1.49–2.62)** |
| Higher degree |  | |  | 0.96 (0.66–1.38) | 0.95 (0.66–1.38) |
| **Housing tenure *(ref. Own home)*** |  | |  |  |  |
| Private rental |  | |  | **1.85 (1.52–2.26)** | **1.85 (1.52–2.27)** |
| Public rental |  | |  | **3.56 (2.58–4.90)** | **3.57 (2.58–4.94)** |
| Other |  | |  | 0.94 (0.51–1.74) | 0.92 (0.49–1.72) |
| **Family structure *(ref. Two-parent)*** |  | |  |  |  |
| Sole parent household |  | |  | **1.45 (1.10–1.90)** | **1.45 (1.10–1.90)** |
| **Maternal age at birth *(ref. 25–35 years)*** |  | |  |  |  |
| Younger than 25 years |  | |  | **0.77 (0.60–0.98)** | **0.76 (0.59–0.97)** |
| Older than 35 years |  | |  | **0.78 (0.62–0.98)** | **0.78 (0.62–0.98)** |
| **Number of children** |  | |  | **1.46 (1.34–1.59)** | **1.46 (1.35–1.59)** |
| **Maternal general health** |  | |  | **0.81 (0.74–0.89)** | **0.81 (0.74–0.89)** |
| **INTERACTIONS** |  | |  |  |  |
| **Benefit 9-months *(ref. no receipt*Time 9-months)*** |  | |  |  |  |
| One benefit*Time 54-months |  | |  |  | **0.52 (0.35–0.78)** |
| Two or more*Time 54-months |  | |  |  | 0.71 (0.41–1.25) |
| One benefit *Time 8-years |  | |  |  | **0.48 (0.31–0.75)** |
| Two or more*Time 8-years |  | |  |  | 0.61 (0.33–1.16) |
| One benefit *Time 12-years |  | |  |  | **0.48 (0.30–0.77)** |
| Two or more*Time 12-years |  | |  |  | 0.98 (0.52–1.86) |
| **Benefit 54-months *(ref. no receipt *Time 9-months)*** |  | |  |  |  |
| One benefit*Time 54-months |  | |  |  | **2.14 (1.40–3.28)** |
| Two or more*Time 54-months |  | |  |  | **2.01 (1.21–3.36)** |
| One benefit*Time 8-years |  | |  |  | 1.06 (0.66–1.70) |
| Two or more*Time 8-years |  | |  |  | **1.91 (1.09–3.35)** |
| One benefit*Time 12 years |  | |  |  | 1.31 (0.79–2.16) |
| Two or more*Time 12 years |  | |  |  | 1.64 (0.91–2.96) |
| **Benefit 8-years *(ref. no receipt *Time 9-months)*** |  | |  |  |  |
| One benefit *Time 54-months |  | |  |  | 1.32 (0.79–2.23) |
| Two or more *Time 54-months |  | |  |  | 1.85 (0.86–3.98) |
| One benefit *Time 8-years |  | |  |  | **2.48 (1.47–4.18)** |
| Two or more *Time 8-years |  | |  |  | **5.64 (2.62–12.15)** |
| One benefit *Time 12 years |  | |  |  | 1.48 (0.84–2.60) |
| Two or more *Time 12 years |  | |  |  | 1.66 (0.73–3.82) |
| **Benefit 12-years *(ref. no receipt *Time 9-months)*** |  | |  |  |  |
| One benefit *Time 54-months |  | |  |  | 0.98 (0.57–1.66) |
| Two or more *Time 54-months |  | |  |  | 1.14 (0.52–2.49) |
| One benefit *Time 8-years |  | |  |  | 1.07 (0.61–1.89) |
| Two or more *Time 8-years |  | |  |  | 0.95 (0.41–2.20) |
| One benefit *Time 12 years |  | |  |  | **2.84 (1.66–4.86)** |
| Two or more *Time 12 years |  | |  |  | **3.48 (1.60–7.58)** |

*Note.* Bolded odds ratio indicates statistical significance at *p* <.05. Ref. = reference group. Continuous scores were converted to z-scores.
